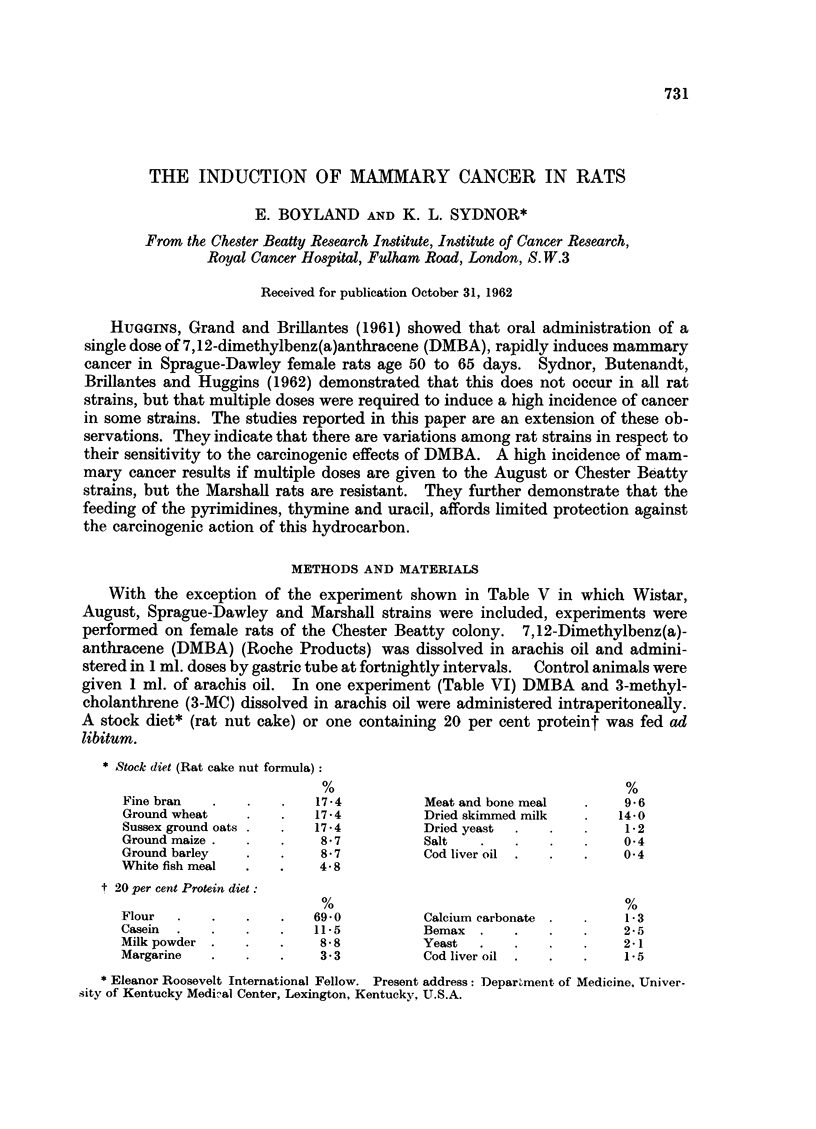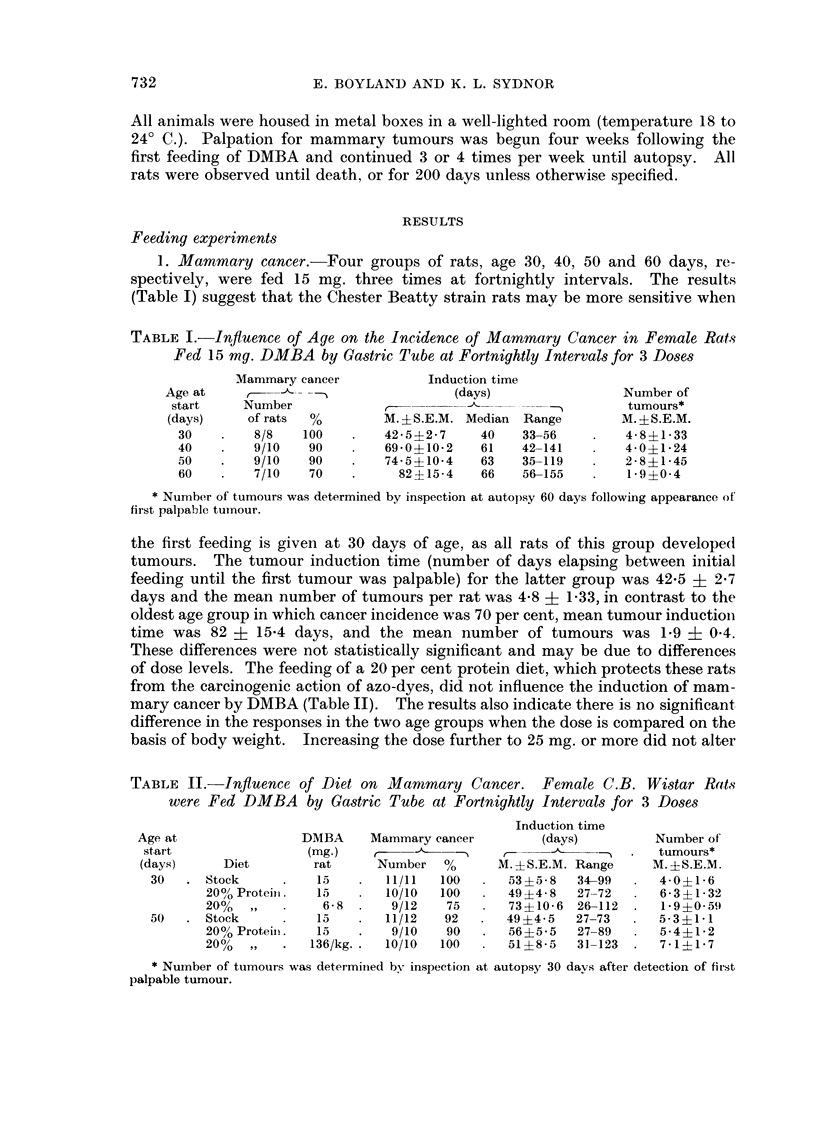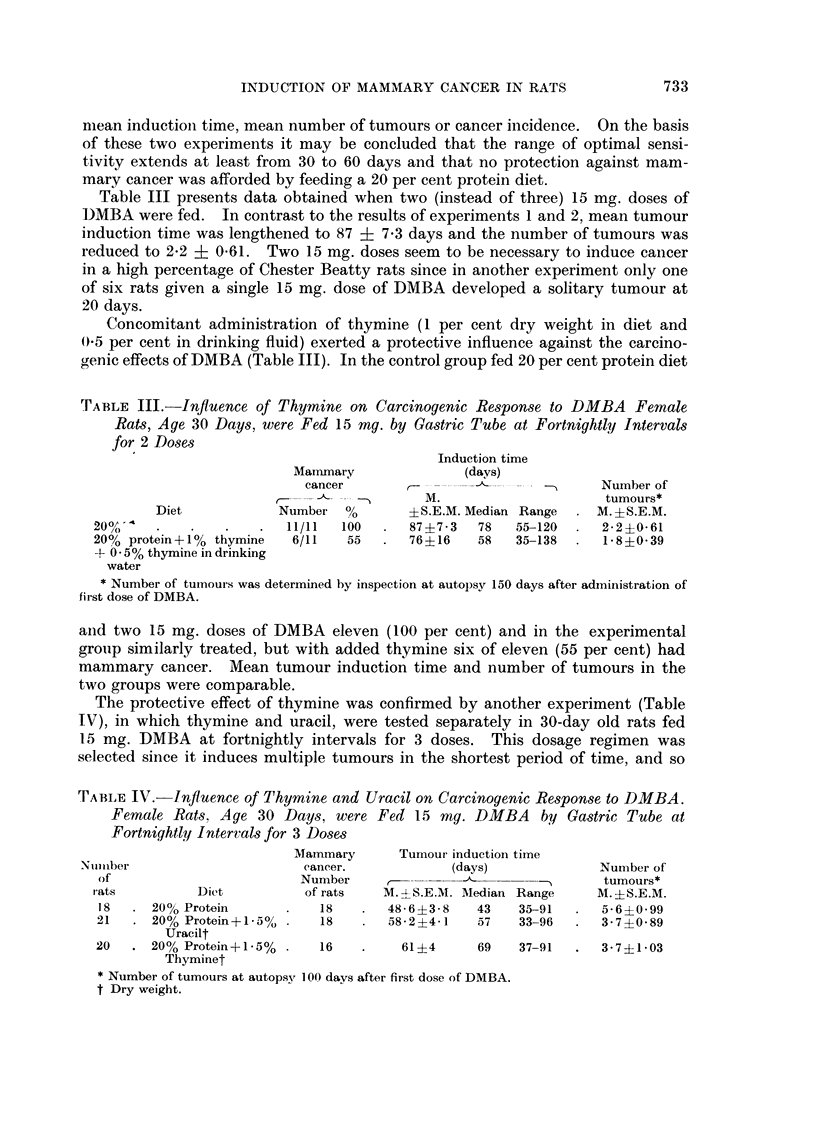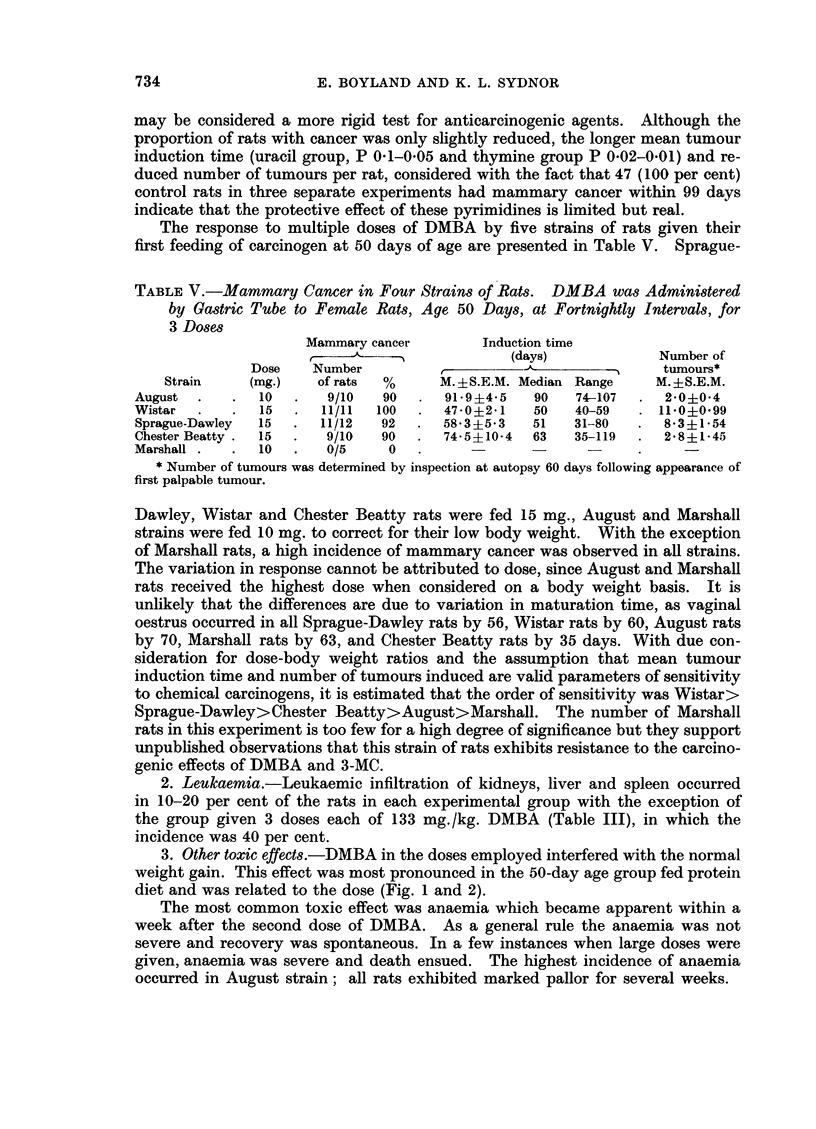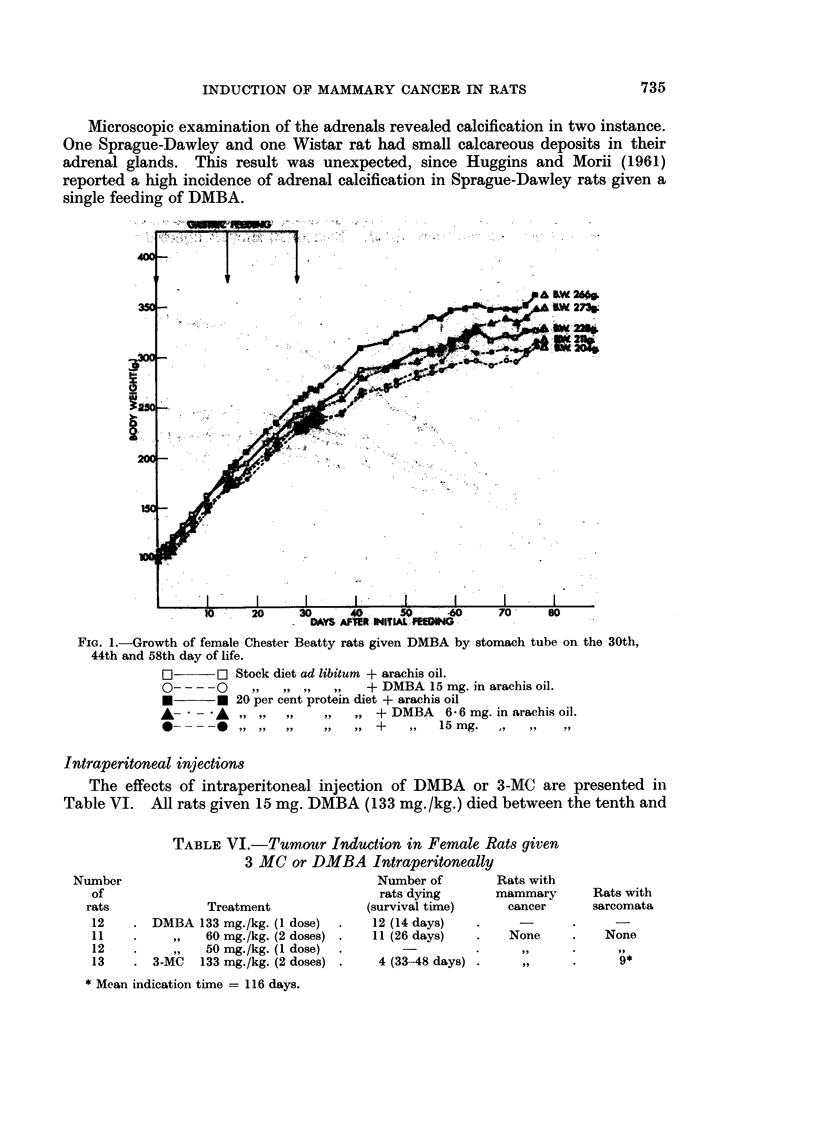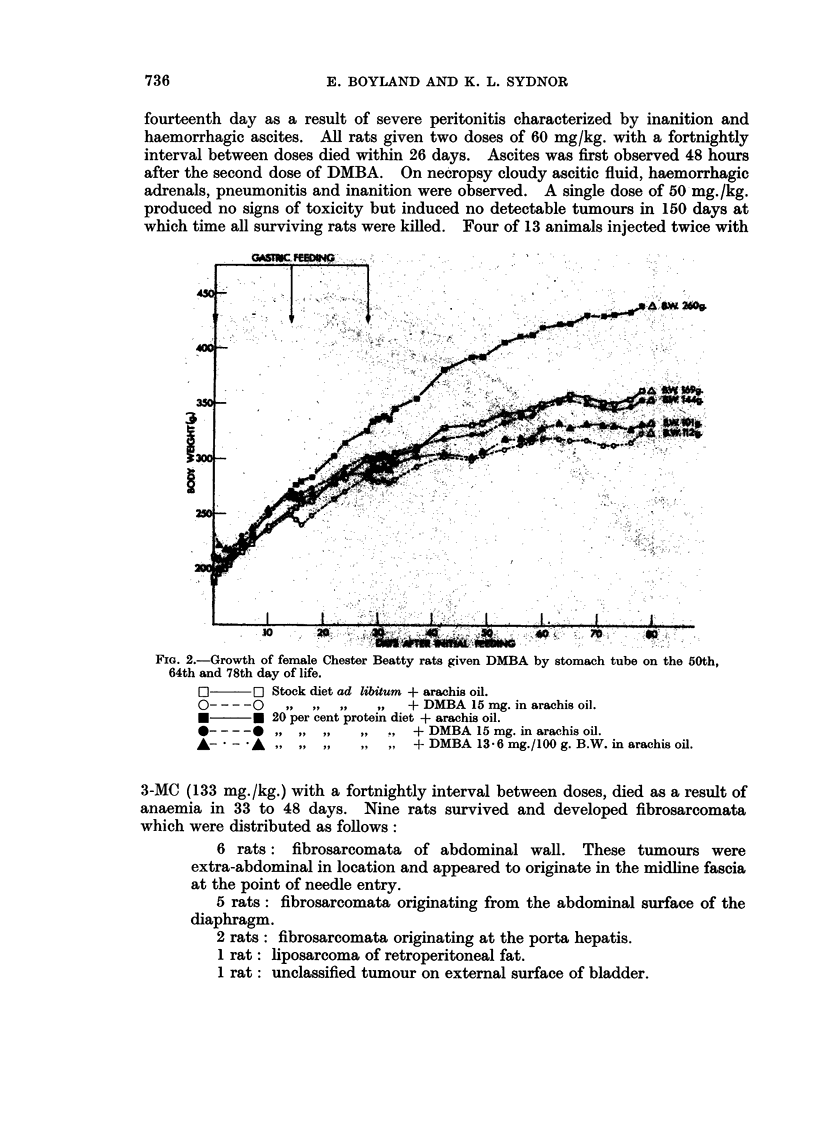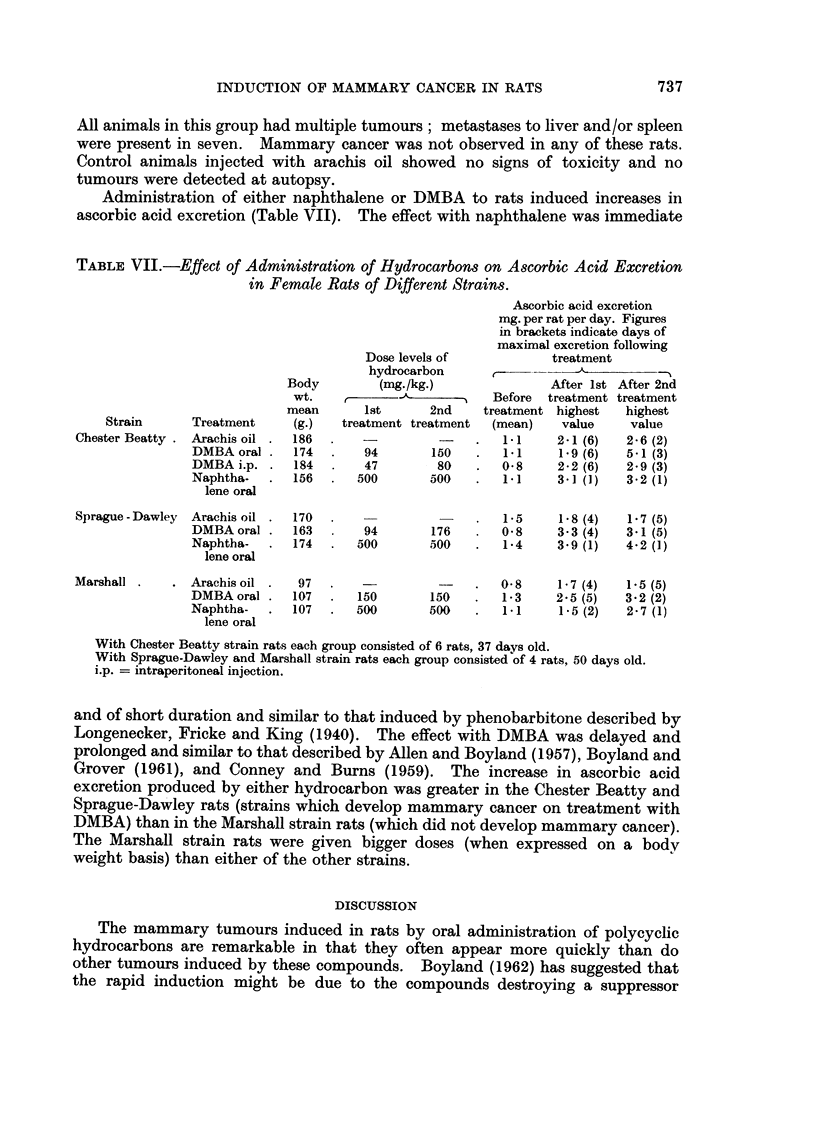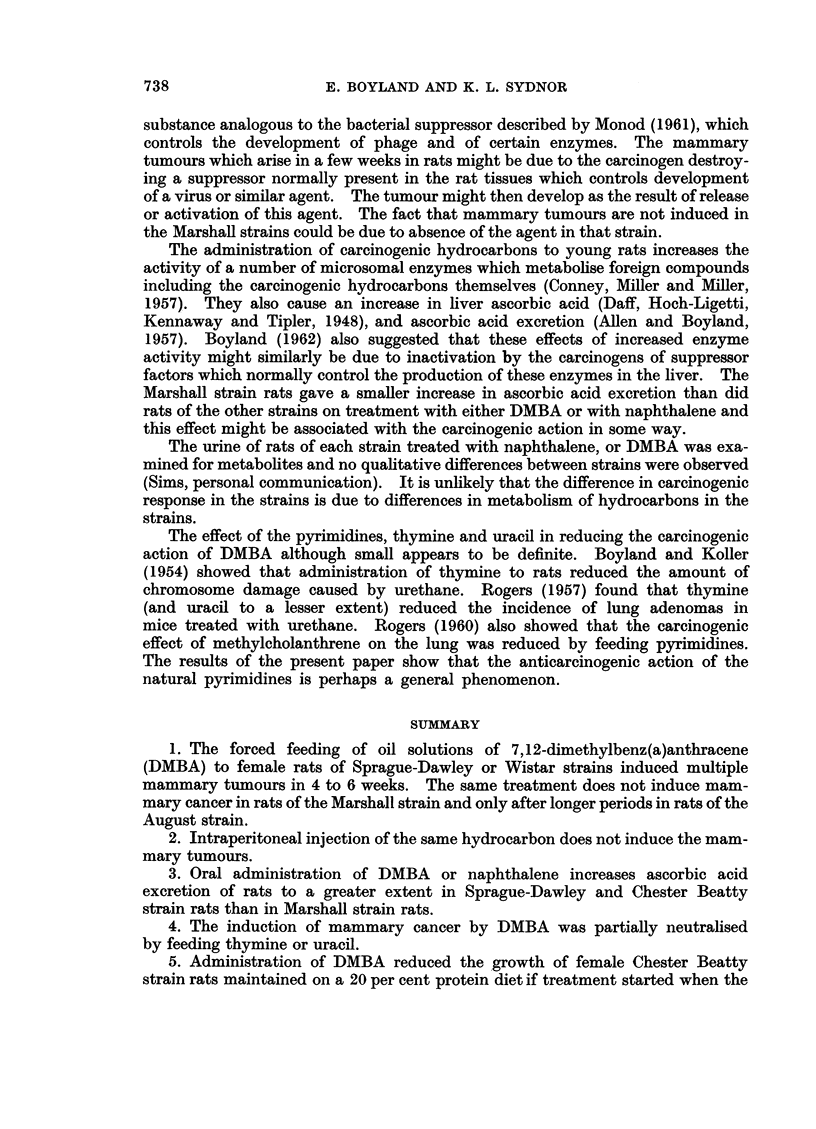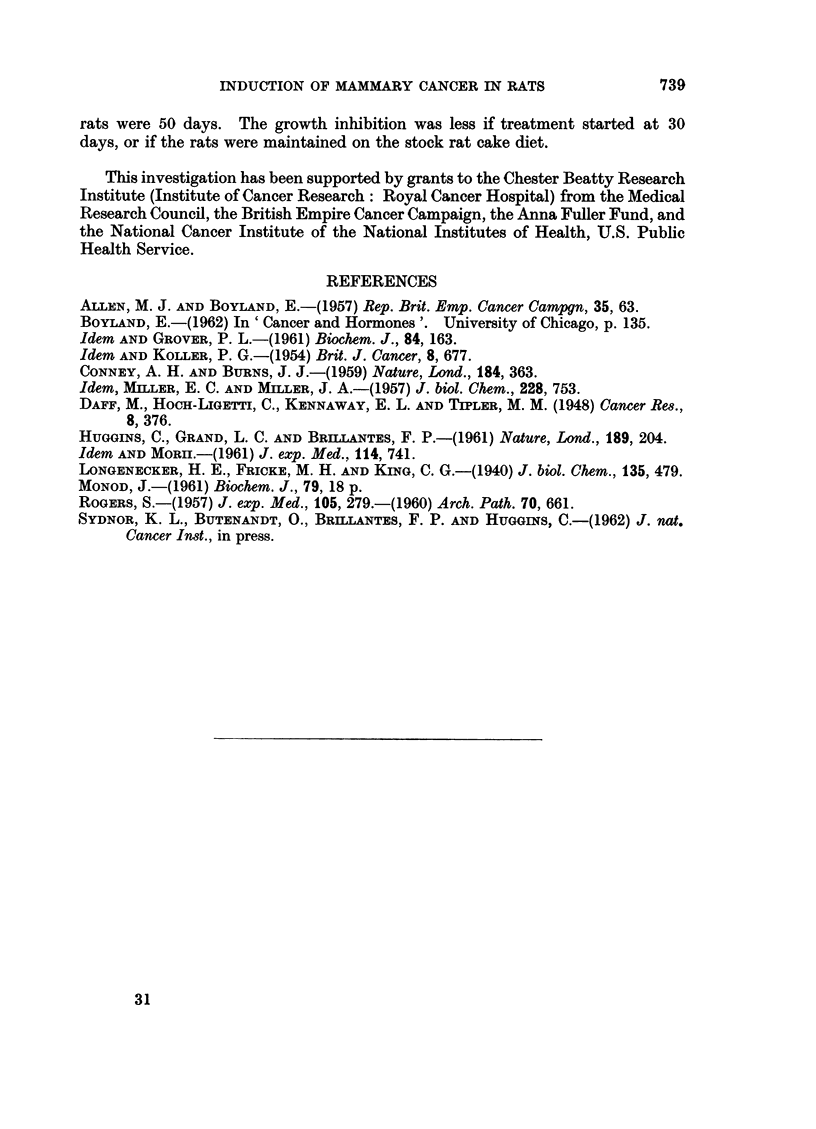# The Induction of Mammary Cancer in Rats

**DOI:** 10.1038/bjc.1962.85

**Published:** 1962-12

**Authors:** E. Boyland, K. L. Sydnor


### THE INDUCTION OF MAMMARY CANCER IN RATS

E. BOYLAND AND K. L. SYDNOR*

*From the Chester Beatty Research Institute, Institute of Cancer Research, Royal Cancer Hospital, Fulham Road, London, S.W.3*

Received for publication October 31, 1962

HUGGINS, Grand and Brillantes (1961) showed that oral administration of a single dose of 7,12-dimethylbenz(a)anthracene (DMBA), rapidly induces mammary cancer in Sprague-Dawley female rats age 50 to 65 days. Sydnor, Butenandt, Brillantes and Huggins (1962) demonstrated that this does not occur in all rat strains, but that multiple doses were required to induce a high incidence of cancer in some strains. The studies reported in this paper are an extension of these observations. They indicate that there are variations among rat strains in respect to their sensitivity to the carcinogenic effects of DMBA. A high incidence of mammary cancer results if multiple doses are given to the August or Chester Beatty strains, but the Marshall rats are resistant. They further demonstrate that the feeding of the pyrimidines, thymine and uracil, affords limited protection against the carcinogenic action of this hydrocarbon.

### METHODS AND MATERIALS

With the exception of the experiment shown in Table V in which Wistar, August, Sprague-Dawley and Marshall strains were included, experiments were performed on female rats of the Chester Beatty colony. 7,12-Dimethylbenz(a)-anthracene (DMBA) (Roche Products) was dissolved in arachis oil and administered in 1 ml. doses by gastric tube at fortnightly intervals. Control animals were given 1 ml. of arachis oil. In one experiment (Table VI) DMBA and 3-methylcholanthrene (3-MC) dissolved in arachis oil were administered intraperitoneally. A stock diet* (rat nut cake) or one containing 20 per cent protein† was fed *ad libitum*.

* *Stock diet* (Rat cake nut formula) :

| | % | | % |
|---|---|---|---|
| Fine bran | 17·4 | Meat and bone meal | 9·6 |
| Ground wheat | 17·4 | Dried skimmed milk | 14·0 |
| Sussex ground oats | 17·4 | Dried yeast | 1·2 |
| Ground maize | 8·7 | Salt | 0·4 |
| Ground barley | 8·7 | Cod liver oil | 0·4 |
| White fish meal | 4·8 | | |

† 20 *per cent Protein diet :*

| | % | | % |
|---|---|---|---|
| Flour | 69·0 | Calcium carbonate | 1·3 |
| Casein | 11·5 | Bemax | 2·5 |
| Milk powder | 8·8 | Yeast | 2·1 |
| Margarine | 3·3 | Cod liver oil | 1·5 |

* Eleanor Roosevelt International Fellow. Present address: Department of Medicine, University of Kentucky Medical Center, Lexington, Kentucky, U.S.A.

All animals were housed in metal boxes in a well-lighted room (temperature 18 to 24° C.). Palpation for mammary tumours was begun four weeks following the first feeding of DMBA and continued 3 or 4 times per week until autopsy. All rats were observed until death, or for 200 days unless otherwise specified.

### RESULTS

### *Feeding experiments*

1. *Mammary cancer.*—Four groups of rats, age 30, 40, 50 and 60 days, respectively, were fed 15 mg. three times at fortnightly intervals. The results (Table I) suggest that the Chester Beatty strain rats may be more sensitive when

TABLE I.—*Influence of Age on the Incidence of Mammary Cancer in Female Rats Fed 15 mg. DMBA by Gastric Tube at Fortnightly Intervals for 3 Doses*

| Age at start (days) | Mammary cancer: Number of rats | % | Induction time (days): M. ± S.E.M. | Median | Range | Number of tumours* M. ± S.E.M. |
|---|---|---|---|---|---|---|
| 30 | 8/8 | 100 | 42·5 ± 2·7 | 40 | 33–56 | 4·8 ± 1·33 |
| 40 | 9/10 | 90 | 69·0 ± 10·2 | 61 | 42–141 | 4·0 ± 1·24 |
| 50 | 9/10 | 90 | 74·5 ± 10·4 | 63 | 35–119 | 2·8 ± 1·45 |
| 60 | 7/10 | 70 | 82 ± 15·4 | 66 | 56–155 | 1·9 ± 0·4 |

* Number of tumours was determined by inspection at autopsy 60 days following appearance of first palpable tumour.

the first feeding is given at 30 days of age, as all rats of this group developed tumours. The tumour induction time (number of days elapsing between initial feeding until the first tumour was palpable) for the latter group was 42·5 ± 2·7 days and the mean number of tumours per rat was 4·8 ± 1·33, in contrast to the oldest age group in which cancer incidence was 70 per cent, mean tumour induction time was 82 ± 15·4 days, and the mean number of tumours was 1·9 ± 0·4. These differences were not statistically significant and may be due to differences of dose levels. The feeding of a 20 per cent protein diet, which protects these rats from the carcinogenic action of azo-dyes, did not influence the induction of mammary cancer by DMBA (Table II). The results also indicate there is no significant difference in the responses in the two age groups when the dose is compared on the basis of body weight. Increasing the dose further to 25 mg. or more did not alter

TABLE II.—*Influence of Diet on Mammary Cancer. Female C.B. Wistar Rats were Fed DMBA by Gastric Tube at Fortnightly Intervals for 3 Doses*

| Age at start (days) | Diet | DMBA (mg.) rat | Mammary cancer: Number | % | Induction time (days): M. ± S.E.M. | Range | Number of tumours* M. ± S.E.M. |
|---|---|---|---|---|---|---|---|
| 30 | Stock | 15 | 11/11 | 100 | 53 ± 5·8 | 34–99 | 4·0 ± 1·6 |
| | 20% Protein | 15 | 10/10 | 100 | 49 ± 4·8 | 27–72 | 6·3 ± 1·32 |
| | 20% ,, | 6·8 | 9/12 | 75 | 73 ± 10·6 | 26–112 | 1·9 ± 0·59 |
| 50 | Stock | 15 | 11/12 | 92 | 49 ± 4·5 | 27–73 | 5·3 ± 1·1 |
| | 20% Protein | 15 | 9/10 | 90 | 56 ± 5·5 | 27–89 | 5·4 ± 1·2 |
| | 20% ,, | 136/kg. | 10/10 | 100 | 51 ± 8·5 | 31–123 | 7·1 ± 1·7 |

* Number of tumours was determined by inspection at autopsy 30 days after detection of first palpable tumour.

mean induction time, mean number of tumours or cancer incidence. On the basis of these two experiments it may be concluded that the range of optimal sensitivity extends at least from 30 to 60 days and that no protection against mammary cancer was afforded by feeding a 20 per cent protein diet.

Table III presents data obtained when two (instead of three) 15 mg. doses of DMBA were fed. In contrast to the results of experiments 1 and 2, mean tumour induction time was lengthened to 87 $\pm$ 7·3 days and the number of tumours was reduced to 2·2 $\pm$ 0·61. Two 15 mg. doses seem to be necessary to induce cancer in a high percentage of Chester Beatty rats since in another experiment only one of six rats given a single 15 mg. dose of DMBA developed a solitary tumour at 20 days.

Concomitant administration of thymine (1 per cent dry weight in diet and 0·5 per cent in drinking fluid) exerted a protective influence against the carcinogenic effects of DMBA (Table III). In the control group fed 20 per cent protein diet

TABLE III.—*Influence of Thymine on Carcinogenic Response to DMBA Female Rats, Age* 30 *Days, were Fed* 15 *mg. by Gastric Tube at Fortnightly Intervals for* 2 *Doses*

| Diet | Mammary cancer: Number | % | Induction time (days): M. ±S.E.M. | Median | Range | Number of tumours* M. ±S.E.M. |
|---|---|---|---|---|---|---|
| 20% | 11/11 | 100 | 87±7·3 | 78 | 55–120 | 2·2±0·61 |
| 20% protein + 1% thymine + 0·5% thymine in drinking water | 6/11 | 55 | 76±16 | 58 | 35–138 | 1·8±0·39 |

* Number of tumours was determined by inspection at autopsy 150 days after administration of first dose of DMBA.

and two 15 mg. doses of DMBA eleven (100 per cent) and in the experimental group similarly treated, but with added thymine six of eleven (55 per cent) had mammary cancer. Mean tumour induction time and number of tumours in the two groups were comparable.

The protective effect of thymine was confirmed by another experiment (Table IV), in which thymine and uracil, were tested separately in 30-day old rats fed 15 mg. DMBA at fortnightly intervals for 3 doses. This dosage regimen was selected since it induces multiple tumours in the shortest period of time, and so

TABLE IV.—*Influence of Thymine and Uracil on Carcinogenic Response to DMBA. Female Rats, Age* 30 *Days, were Fed* 15 *mg. DMBA by Gastric Tube at Fortnightly Intervals for* 3 *Doses*

| Number of rats | Diet | Mammary cancer. Number of rats | Tumour induction time (days): M. ±S.E.M. | Median | Range | Number of tumours* M. ±S.E.M. |
|---|---|---|---|---|---|---|
| 18 | 20% Protein | 18 | 48·6±3·8 | 43 | 35–91 | 5·6±0·99 |
| 21 | 20% Protein + 1·5% Uracil† | 18 | 58·2±4·1 | 57 | 33–96 | 3·7±0·89 |
| 20 | 20% Protein + 1·5% Thymine† | 16 | 61±4 | 69 | 37–91 | 3·7±1·03 |

* Number of tumours at autopsy 100 days after first dose of DMBA.
† Dry weight.

may be considered a more rigid test for anticarcinogenic agents. Although the proportion of rats with cancer was only slightly reduced, the longer mean tumour induction time (uracil group, P 0·1–0·05 and thymine group P 0·02–0·01) and reduced number of tumours per rat, considered with the fact that 47 (100 per cent) control rats in three separate experiments had mammary cancer within 99 days indicate that the protective effect of these pyrimidines is limited but real.

The response to multiple doses of DMBA by five strains of rats given their first feeding of carcinogen at 50 days of age are presented in Table V. Sprague-Dawley, Wistar and Chester Beatty rats were fed 15 mg., August and Marshall strains were fed 10 mg. to correct for their low body weight. With the exception of Marshall rats, a high incidence of mammary cancer was observed in all strains. The variation in response cannot be attributed to dose, since August and Marshall rats received the highest dose when considered on a body weight basis. It is unlikely that the differences are due to variation in maturation time, as vaginal oestrus occurred in all Sprague-Dawley rats by 56, Wistar rats by 60, August rats by 70, Marshall rats by 63, and Chester Beatty rats by 35 days. With due consideration for dose-body weight ratios and the assumption that mean tumour induction time and number of tumours induced are valid parameters of sensitivity to chemical carcinogens, it is estimated that the order of sensitivity was Wistar> Sprague-Dawley>Chester Beatty>August>Marshall. The number of Marshall rats in this experiment is too few for a high degree of significance but they support unpublished observations that this strain of rats exhibits resistance to the carcinogenic effects of DMBA and 3-MC.

TABLE V.—*Mammary Cancer in Four Strains of Rats. DMBA was Administered by Gastric Tube to Female Rats, Age* 50 *Days, at Fortnightly Intervals, for* 3 *Doses*

| Strain | Dose (mg.) | Mammary cancer: Number of rats | Mammary cancer: % | Induction time (days): M. ± S.E.M. | Induction time (days): Median | Induction time (days): Range | Number of tumours* M. ± S.E.M. |
|---|---|---|---|---|---|---|---|
| August | 10 | 9/10 | 90 | 91·9 ± 4·5 | 90 | 74–107 | 2·0 ± 0·4 |
| Wistar | 15 | 11/11 | 100 | 47·0 ± 2·1 | 50 | 40–59 | 11·0 ± 0·99 |
| Sprague-Dawley | 15 | 11/12 | 92 | 58·3 ± 5·3 | 51 | 31–80 | 8·3 ± 1·54 |
| Chester Beatty | 15 | 9/10 | 90 | 74·5 ± 10·4 | 63 | 35–119 | 2·8 ± 1·45 |
| Marshall | 10 | 0/5 | 0 | — | — | — | — |

* Number of tumours was determined by inspection at autopsy 60 days following appearance of first palpable tumour.

2. *Leukaemia.*—Leukaemic infiltration of kidneys, liver and spleen occurred in 10–20 per cent of the rats in each experimental group with the exception of the group given 3 doses each of 133 mg./kg. DMBA (Table III), in which the incidence was 40 per cent.

3. *Other toxic effects.*—DMBA in the doses employed interfered with the normal weight gain. This effect was most pronounced in the 50-day age group fed protein diet and was related to the dose (Fig. 1 and 2).

The most common toxic effect was anaemia which became apparent within a week after the second dose of DMBA. As a general rule the anaemia was not severe and recovery was spontaneous. In a few instances when large doses were given, anaemia was severe and death ensued. The highest incidence of anaemia occurred in August strain ; all rats exhibited marked pallor for several weeks.

Microscopic examination of the adrenals revealed calcification in two instance. One Sprague-Dawley and one Wistar rat had small calcareous deposits in their adrenal glands. This result was unexpected, since Huggins and Morii (1961) reported a high incidence of adrenal calcification in Sprague-Dawley rats given a single feeding of DMBA.

FIG. 1.—Growth of female Chester Beatty rats given DMBA by stomach tube on the 30th, 44th and 58th day of life.

□———□ Stock diet *ad libitum* + arachis oil.
○- - - -○ ,, ,, ,, ,, + DMBA 15 mg. in arachis oil.
■———■ 20 per cent protein diet + arachis oil
▲- · - ·▲ ,, ,, ,, ,, ,, + DMBA 6·6 mg. in arachis oil.
●- - - -● ,, ,, ,, ,, ,, + ,, 15 mg. ,, ,, ,,

*Intraperitoneal injections*

The effects of intraperitoneal injection of DMBA or 3-MC are presented in Table VI. All rats given 15 mg. DMBA (133 mg./kg.) died between the tenth and

TABLE VI.—*Tumour Induction in Female Rats given 3 MC or DMBA Intraperitoneally*

| Number of rats | Treatment | Number of rats dying (survival time) | Rats with mammary cancer | Rats with sarcomata |
|---|---|---|---|---|
| 12 | DMBA 133 mg./kg. (1 dose) | 12 (14 days) | — | — |
| 11 | ,, 60 mg./kg. (2 doses) | 11 (26 days) | None | None |
| 12 | ,, 50 mg./kg. (1 dose) | — | ,, | ,, |
| 13 | 3-MC 133 mg./kg. (2 doses) | 4 (33–48 days) | ,, | 9* |

* Mean indication time = 116 days.

fourteenth day as a result of severe peritonitis characterized by inanition and haemorrhagic ascites. All rats given two doses of 60 mg/kg. with a fortnightly interval between doses died within 26 days. Ascites was first observed 48 hours after the second dose of DMBA. On necropsy cloudy ascitic fluid, haemorrhagic adrenals, pneumonitis and inanition were observed. A single dose of 50 mg./kg. produced no signs of toxicity but induced no detectable tumours in 150 days at which time all surviving rats were killed. Four of 13 animals injected twice with

FIG. 2.—Growth of female Chester Beatty rats given DMBA by stomach tube on the 50th, 64th and 78th day of life.

□———□ Stock diet *ad libitum* + arachis oil.

○- - - -○ „ „ „ „ + DMBA 15 mg. in arachis oil.

■———■ 20 per cent protein diet + arachis oil.

●- - - -● „ „ „ „ „ + DMBA 15 mg. in arachis oil.

▲- · - ·▲ „ „ „ „ „ + DMBA 13·6 mg./100 g. B.W. in arachis oil.

3-MC (133 mg./kg.) with a fortnightly interval between doses, died as a result of anaemia in 33 to 48 days. Nine rats survived and developed fibrosarcomata which were distributed as follows :

6 rats : fibrosarcomata of abdominal wall. These tumours were extra-abdominal in location and appeared to originate in the midline fascia at the point of needle entry.

5 rats : fibrosarcomata originating from the abdominal surface of the diaphragm.

2 rats : fibrosarcomata originating at the porta hepatis.

1 rat : liposarcoma of retroperitoneal fat.

1 rat : unclassified tumour on external surface of bladder.

All animals in this group had multiple tumours ; metastases to liver and/or spleen were present in seven. Mammary cancer was not observed in any of these rats. Control animals injected with arachis oil showed no signs of toxicity and no tumours were detected at autopsy.

Administration of either naphthalene or DMBA to rats induced increases in ascorbic acid excretion (Table VII). The effect with naphthalene was immediate

TABLE VII.—*Effect of Administration of Hydrocarbons on Ascorbic Acid Excretion in Female Rats of Different Strains.*

| Strain | Treatment | Body wt. mean (g.) | Dose levels of hydrocarbon (mg./kg.) 1st treatment | 2nd treatment | Ascorbic acid excretion mg. per rat per day. Figures in brackets indicate days of maximal excretion following treatment: Before treatment (mean) | After 1st treatment highest value | After 2nd treatment highest value |
|---|---|---|---|---|---|---|---|
| Chester Beatty | Arachis oil | 186 | — | — | 1·1 | 2·1 (6) | 2·6 (2) |
| | DMBA oral | 174 | 94 | 150 | 1·1 | 1·9 (6) | 5·1 (3) |
| | DMBA i.p. | 184 | 47 | 80 | 0·8 | 2·2 (6) | 2·9 (3) |
| | Naphthalene oral | 156 | 500 | 500 | 1·1 | 3·1 (1) | 3·2 (1) |
| Sprague - Dawley | Arachis oil | 170 | — | — | 1·5 | 1·8 (4) | 1·7 (5) |
| | DMBA oral | 163 | 94 | 176 | 0·8 | 3·3 (4) | 3·1 (5) |
| | Naphthalene oral | 174 | 500 | 500 | 1·4 | 3·9 (1) | 4·2 (1) |
| Marshall | Arachis oil | 97 | — | — | 0·8 | 1·7 (4) | 1·5 (5) |
| | DMBA oral | 107 | 150 | 150 | 1·3 | 2·5 (5) | 3·2 (2) |
| | Naphthalene oral | 107 | 500 | 500 | 1·1 | 1·5 (2) | 2·7 (1) |

With Chester Beatty strain rats each group consisted of 6 rats, 37 days old.
With Sprague-Dawley and Marshall strain rats each group consisted of 4 rats, 50 days old.
i.p. = intraperitoneal injection.

and of short duration and similar to that induced by phenobarbitone described by Longenecker, Fricke and King (1940). The effect with DMBA was delayed and prolonged and similar to that described by Allen and Boyland (1957), Boyland and Grover (1961), and Conney and Burns (1959). The increase in ascorbic acid excretion produced by either hydrocarbon was greater in the Chester Beatty and Sprague-Dawley rats (strains which develop mammary cancer on treatment with DMBA) than in the Marshall strain rats (which did not develop mammary cancer). The Marshall strain rats were given bigger doses (when expressed on a body weight basis) than either of the other strains.

### DISCUSSION

The mammary tumours induced in rats by oral administration of polycyclic hydrocarbons are remarkable in that they often appear more quickly than do other tumours induced by these compounds. Boyland (1962) has suggested that the rapid induction might be due to the compounds destroying a suppressor substance analogous to the bacterial suppressor described by Monod (1961), which controls the development of phage and of certain enzymes. The mammary tumours which arise in a few weeks in rats might be due to the carcinogen destroying a suppressor normally present in the rat tissues which controls development of a virus or similar agent. The tumour might then develop as the result of release or activation of this agent. The fact that mammary tumours are not induced in the Marshall strains could be due to absence of the agent in that strain.

The administration of carcinogenic hydrocarbons to young rats increases the activity of a number of microsomal enzymes which metabolise foreign compounds including the carcinogenic hydrocarbons themselves (Conney, Miller and Miller, 1957). They also cause an increase in liver ascorbic acid (Daff, Hoch-Ligetti, Kennaway and Tipler, 1948), and ascorbic acid excretion (Allen and Boyland, 1957). Boyland (1962) also suggested that these effects of increased enzyme activity might similarly be due to inactivation by the carcinogens of suppressor factors which normally control the production of these enzymes in the liver. The Marshall strain rats gave a smaller increase in ascorbic acid excretion than did rats of the other strains on treatment with either DMBA or with naphthalene and this effect might be associated with the carcinogenic action in some way.

The urine of rats of each strain treated with naphthalene, or DMBA was examined for metabolites and no qualitative differences between strains were observed (Sims, personal communication). It is unlikely that the difference in carcinogenic response in the strains is due to differences in metabolism of hydrocarbons in the strains.

The effect of the pyrimidines, thymine and uracil in reducing the carcinogenic action of DMBA although small appears to be definite. Boyland and Koller (1954) showed that administration of thymine to rats reduced the amount of chromosome damage caused by urethane. Rogers (1957) found that thymine (and uracil to a lesser extent) reduced the incidence of lung adenomas in mice treated with urethane. Rogers (1960) also showed that the carcinogenic effect of methylcholanthrene on the lung was reduced by feeding pyrimidines. The results of the present paper show that the anticarcinogenic action of the natural pyrimidines is perhaps a general phenomenon.

### SUMMARY

1. The forced feeding of oil solutions of 7,12-dimethylbenz(a)anthracene (DMBA) to female rats of Sprague-Dawley or Wistar strains induced multiple mammary tumours in 4 to 6 weeks. The same treatment does not induce mammary cancer in rats of the Marshall strain and only after longer periods in rats of the August strain.

2. Intraperitoneal injection of the same hydrocarbon does not induce the mammary tumours.

3. Oral administration of DMBA or naphthalene increases ascorbic acid excretion of rats to a greater extent in Sprague-Dawley and Chester Beatty strain rats than in Marshall strain rats.

4. The induction of mammary cancer by DMBA was partially neutralised by feeding thymine or uracil.

5. Administration of DMBA reduced the growth of female Chester Beatty strain rats maintained on a 20 per cent protein diet if treatment started when the rats were 50 days. The growth inhibition was less if treatment started at 30 days, or if the rats were maintained on the stock rat cake diet.

This investigation has been supported by grants to the Chester Beatty Research Institute (Institute of Cancer Research : Royal Cancer Hospital) from the Medical Research Council, the British Empire Cancer Campaign, the Anna Fuller Fund, and the National Cancer Institute of the National Institutes of Health, U.S. Public Health Service.

### REFERENCES

Allen, M. J. and Boyland, E.—(1957) *Rep. Brit. Emp. Cancer Campgn*, **35**, 63.
Boyland, E.—(1962) In ' Cancer and Hormones '. University of Chicago, p. 135.
*Idem* and Grover, P. L.—(1961) *Biochem. J.*, **84**, 163.
*Idem* and Koller, P. G.—(1954) *Brit. J. Cancer*, **8**, 677.
Conney, A. H. and Burns, J. J.—(1959) *Nature, Lond.*, **184**, 363.
*Idem*, Miller, E. C. and Miller, J. A.—(1957) *J. biol. Chem.*, **228**, 753.
Daff, M., Hoch-Ligetti, C., Kennaway, E. L. and Tipler, M. M. (1948) *Cancer Res.*, **8**, 376.
Huggins, C., Grand, L. C. and Brillantes, F. P.—(1961) *Nature, Lond.*, **189**, 204.
*Idem* and Morii.—(1961) *J. exp. Med.*, **114**, 741.
Longenecker, H. E., Fricke, M. H. and King, C. G.—(1940) *J. biol. Chem.*, **135**, 479.
Monod, J.—(1961) *Biochem. J.*, **79**, 18 p.
Rogers, S.—(1957) *J. exp. Med.*, **105**, 279.—(1960) *Arch. Path.* **70**, 661.
Sydnor, K. L., Butenandt, O., Brillantes, F. P. and Huggins, C.—(1962) *J. nat. Cancer Inst.*, in press.